# A Survey of the Perceived Risk for Stroke among Community Residents in Western Urban China

**DOI:** 10.1371/journal.pone.0073578

**Published:** 2013-09-11

**Authors:** Juan Yang, Min Zheng, Shuqun Chen, Shu Ou, Jie Zhang, Ni Wang, Yingying Cao, Qiaoqiao Miao, Xingxiu Zhang, Ling Hao, Jinhe Lou, Huijuan Guo, Nan Li, Jian Wang

**Affiliations:** 1 Department of Neurology, the Second Affiliated Hospital, Chongqing Medical University, Chongqing, China; 2 Basic Medical College, Chongqing Medical University, Chongqing, China; 3 Department of Preventive Medicine, Chongqing Medical University, Chongqing, China; Institute of Neuroepidemiology and Tropical Neurology, France

## Abstract

**Background and Purpose:**

Persons who perceive their risk for stroke can promote the intervention of stroke risk factors and reduce the risk of stroke occurrence. Our purpose was to assess the knowledge of stroke risk factors and the level of perceived risk for stroke.

**Methods:**

In 2011, a population-based face-to-face interview survey was conducted in Yuzhong district, Chongqing. A total of 1500 potential participants aged ≥18 years old were selected using a multi-stage sampling method. The knowledge of stroke risk factors and perceived risk for stroke was surveyed.

**Results:**

A total of 941 participants completed the questionnaire survey. The respondents’ awareness rate of stroke risk factors ranged between 53.3% and 87.2%. The community residents’ perceived risk for stroke was only 17.7%. Multiple logistic regression analysis showed that 45–64 years age group, a history of hypertension, hyperlipidemia, heart disease, and stroke were independent predictors of perceived risk for stroke. Perceived risk for stroke increased as the number of risk factors increased (*P*<0.001). However, even for respondents with three or more risk factors, only 41% perceived themselves to be at risk for stroke.

**Conclusions:**

In this population-based survey, few community residents perceived risk for stroke, even among those with multiple stroke risk factors, most did not perceive themselves to be at risk for stroke. Persons with 45–64 years old, a history of hypertension, hyperlipidemia, heart disease or stroke were more likely to perceive risk for stroke. The awareness of the risk for stroke has yet to be enhanced among community residents.

## Introduction

Currently, cerebrovascular diseases have become the third leading cause of death worldwide and the leading cause of adult disability [1]. It has become the leading cause of death in China [2], with the mortality estimated at more than 1.5 million per year. According to the world health organization survey, the incidence of stroke in China is higher than the world average level, and it is still rising at an annual rate of 8.7% [3]. The common stroke risk factors have a high prevalence in China. The recent survey showed that the prevalence of hypertension, diabetes and hyperlipidemia was 18.8%, 2.6% and 18.6% respectively [4]. Numerous studies have shown that the intervention of stroke risk factors can reduce the incidence of stroke [5]–[8]. Moreover, the perceived risk for stroke can promote the intervention of risk factors, thus reducing the risk of stroke occurrence [9]. Few population-based studies have found that most patients did not consider themselves to be at risk for stroke, even among people with multiple risk factors, the level of perceived risk for stroke is still very low [10]. Currently, there is a lack of similar research reports in China. In 2011, we surveyed the knowledge of stroke risk factors and the perceived risk for stroke among residents of Yuzhong district, Chongqing, and analyzed the relationship between the two characters.

## Methods

### Setting and Sampling

**Figure 1:**
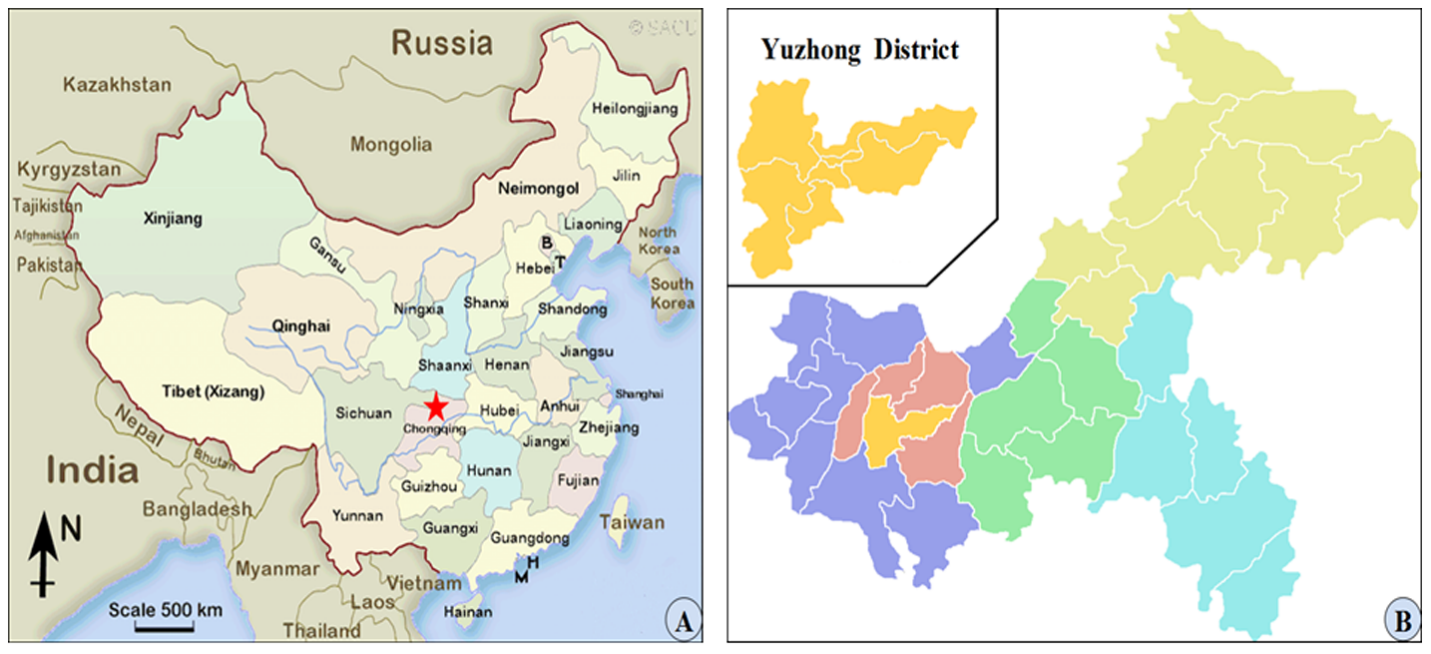
Map of study location. A, Chongqing is located in western China (red pentagram). B, The urban area of Chongqing municipality includes nine districts. Yuzhong District is the central (yellow) and most densely populated district. There are 12 blocks and about 660,000 permanent population in Yuzhong district.

This is a cross-sectional study. According to the method of estimated the minimum sample size of qualitative data recommended by Chinese Residents of Nutrition and Health Survey in 2002, 1500 households in Yuzhong district, the center of Chongqing with a permanent population of 660,000 and 12 blocks (Figure 1), were randomly selected between March 20 and August 23, 2011. A multi-stage sampling method was adopted. First, cluster sampling was conducted to sample three blocks from Yuzhong district and five communities were randomly selected in each block, then systematic sampling was conducted to sample 100 households in each community according to the residence number. Within each household, persons who were eligible to participate in the study must be at least 18 years of age, lived for more than 2 years, and had no mental disease or cognitive dysfunction which could lead to noncooperate with the interview. Afterwards, a random-number grid was used to select one eligible resident to be surveyed.

### Survey Content and Diagnosis Criteria

We reviewed some literatures about stroke risk factors and perceived risk for stroke [9]–[13], and then developed a self-designed questionnaire. It was pretested with 50 people to detect potential problems. The final revised questionnaire contained 4 sections: (1) Respondents’ demographic details such as gender, age, ethnicity, educational level, monthly household income, and health insurance. (2) The presence of stroke risk factors (smoking, history of hypertension, diabetes, hyperlipidemia, heart disease and previous stroke) was based on self-report. Respondents were asked “Have you ever been told by a physician or nurse that you have: (risk factors)”. Respondents who reported that they have smoked one or more cigarettes everyday in general for more than 6 consecutive months and smoked within one month before survey were categorized as current smokers. Non-smoker was defined as never smoking, smoking but did not meet the above criteria, or had history of smoking but had quitted smoking for at least 6 consecutive months [14]. Hypertension was defined as a mean systolic blood pressure ≥140 mmHg and/or a mean diastolic blood pressure ≥90 mmHg, or if the subject was currently receiving antihypertensive treatment [15]. Diabetes was defined as glycated hemoglobin A1c ≥6.5%, or the fasting blood glucose (FPG) ≥7.0 mmol/L, or 2 h oral glucose tolerance test glucose ≥11.1 mmol/L, or random blood glucose ≥11.1 mmol/L with typical symptoms of high blood sugar [16]. Hyperlipidemia was defined as total cholesterol (TC) >5.18 mmol/L, low-density lipoprotein cholesterol (LDL-C) >3.37 mmol/L, or triglyceride (TG) >1.70 mmol/L [17]. Respondents who reported a history of a coronary heart disease, hypertensive heart disease, atrial fibrillation, or rheumatic heart disease were classified as having a history of heart disease [18]. At the beginning of the survey, the concept of stroke was explained to the respondents as following: stroke, also known as apoplexy or cerebrovascular accident, is rapid loss of brain function due to a blockage or rupture of a blood vessel to the brain. The symptoms include sudden limb weakness, disturbance of sensation, speech disorder, and so on [19]. (3) Respondents were asked the following question to assess their perceived risk for stroke. “Based on your current physical status, do you think you are at risk of having a stroke?” Respondents answered with “yes”, “no”, or “unknown/unsure”. (4) Knowledge of stroke risk factors. Respondents were asked “Do you think the following six factors are stroke risk factors?” The answer for each factor is “yes”, “no”, or “unknown/unsure”. The respondents were asked about stroke risk factors after information was obtained about perceived risk for stroke.

### Data Collection

The interview team made appointment with the selected household about the time and place before survey. After signing the informed consent form, the uniformly trained investigators conducted the survey through face-to-face interview with selected household member at the scheduled time and place. All the data were anonymized.

### Ethical Considerations and Procedure

We had already discussed the ethical issues about the survey with the ethics committee of the second affiliated hospital, Chongqing medical university. Because this survey was an anonymous survey, and did not involve any clinical biological specimen collection (such as blood) with invasive operation, ethics approval was deemed unnecessary. In order to obtain the residents’ understanding and cooperation with the survey, we notified the household in advance to explain the purpose and significance of the survey by issuing an informed consent form. In addition, we informed the household this was an anonymous survey which did not involve the name and contact information, so there would be no leakage of personal privacy.

### Statistical Analysis

All statistical analyses were completed using SPSS11.5 statistical software. Descriptive statistical analysis was used to analyze the demographic characteristics of the respondents. Reliability of the self-questionnaire was evaluated using Cronbach α. Chi-squared tests were used to analyze the univariate relationship among demographic characteristics, self-reported stroke risk factors, knowledge of stroke risk factors and the perceived risk for stroke. Multiple logistic regression analysis was used to identify factors independently associated with perceived risk for stroke among respondents overall and among those with two or more stroke risk factors. *P*<0.05 was considered statistically significant.

## Results

In total, 1,500 households were sampled from the fifteen communities in the three blocks described in the methods. Overall, 972 residents participated in the survey. 941 completed the questionnaire. The response rate was 62.7%. The other 31 questionnaires were excluded for incomplete information. The reliability of the self-questionnaire was good (Cronbach α = 0.78 for presence of stroke risk factors and Cronbach α = 0.82 for knowledge of stroke risk factors). The mean age was 58.6±15.2 years (range, 18 to 91 years). 61.8% were female, 98% were the ethnic Han, 84.1% had health insurance, and 82.9% reported their educational level was lower than college (Table 1).

**Table 1:** Perceived Risk for Stroke among Community Residents.

	N (%) (n = 941)	Stroke Risk		*P* ^a^	AOR (95% CI)^b^
		Yes (n = 166)	No (n = 775)		
Gender				0.265	
Male	359 (38.2)	57 (15.9)	302 (84.1)		0.93 (0.58–1.47)
Female	582 (61.8)	109 (18.7)	473 (81.3)		–
Age Group (years)				<0.001	
18–44	144 (15.3)	8 (5.6)	136 (94.4)		0.65 (0.27–1.55)
45–64	469 (49.8)	101 (21.5)	368 (78.5)		1.89 (1.23–2.93)
≥65	328 (34.9)	57 (17.4)	271 (82.6)		–
Ethnicity				0.928	
Han	922 (98.0)	162 (17.6)	760 (82.4)		0.73 (0.20–2.61)
Other	19 (2.0)	4 (21.1)	15 (78.9)		–
Educational Level				0.052	
Primary School or Below	258 (27.4)	33 (12.8)	225 (87.2)		0.53 (0.27–1.06)
Middle School	291 (30.9)	57 (19.6)	234 (80.4)		1.00 (0.55–1.84)
High School	231 (24.5)	50 (21.6)	181 (78.4)		1.15 (0.64–2.08)
College or Above	161 (17.1)	26 (16.1)	135 (83.9)		–
Monthly Household Income (RMB c)				0.663	
<2000	313 (33.3)	53 (16.9)	260 (83.1)		1.04 (0.52–2.06)
2000–3999	358 (38.0)	59 (16.5)	299 (83.5)		0.86 (0.44–1.66)
4000–7999	175 (18.6)	36 (20.6)	139 (79.4)		0.97 (0.48–1.95)
≥8000	95 (10.1)	18 (18.9)	77 (81.1)		–
Health Insurance				0.048	
Yes	791 (84.1)	148 (18.7)	643 (81.3)		0.92 (0.51–1.66)
No	150 (15.9)	18 (12.0)	132 (88.0)		–
Smoking				0.285	
Current smoker	193 (20.5)	29 (15.0)	164 (85.0)		0.88 (0.50–1.56)
Non-smoker	748 (79.5)	137(18.3)	611 (81.7)		–
Hypertension				<0.001	
Yes	296 (31.5)	84 (28.4)	212 (71.6)		1.73 (1.16–2.59)
No	645 (68.5)	82 (12.7)	563 (87.3)		–
Diabetes				0.122	
Yes	119 (12.6)	27 (22.7)	92 (77.3)		0.92 (0.54–1.57)
No	822 (87.4)	139 (16.9)	683 (83.1)		–
Hyperlipidemia				<0.001	
Yes	177 (18.8)	59 (33.3)	118 (66.7)		1.84 (1.20–2.84)
No	764 (81.2)	107 (14.0)	657 (86.0)		–
Heart Disease				<0.001	
Yes	170 (18.1)	63 (37.1)	107 (62.9)		2.92 (1.88–4.52)
No	771 (81.9)	103 (13.4)	668 (86.6)		–
Previous Stroke				<0.001	
Yes	74 (7.9)	32 (43.2)	42 (56.8)		2.70 (1.55–4.70)
No	867 (92.1)	134 (15.5)	733 (84.5)		–

Notes: ^a^results of a chi-squared test,

b results of multiple logistic regression analysis,

c exchange rate: 100 Euro ≈ 920.07 RMB.

Of the 941 respondents, 31.5% reported that they had been told by a physician or nurse that they had hypertension, 12.6% reported diabetes, 18.8% reported hyperlipidemia, 18.1% reported heart disease, 7.9% reported previous stroke, and 20.5% reported smoking. 62.8% had 1 or more risk factors, 30.7% were 2 or more risk factors, and 14.5% reported 3 or more stroke risk factors.

The respondents’ awareness rate of stroke risk factors ranged between 53.3% and 87.2%. The risk factor that had the highest awareness rate was hypertension (87.2%), followed by hyperlipidemia (74.2%) and previous stroke history (73.4%), smoking (60.9%) and heart disease (60.9%), and finally diabetes (53.3%). Only 27.9% respondents correctly identify all the six listed controllable stroke risk factors, while 4.5% respondents could not identify any of them. In the univariate comparison, higher educational level (*P*<0.001), higher household income (*P*<0.001), with health insurance (*P = *0.021), smoking (*P = *0.049), with hyperlipidemia (*P = *0.014) predicted better knowledge of stroke risk factors. The educational level was independently associated with knowing all of the six stroke risk factors (*P*<0.001). While there was no difference in terms of gender, age group, ethnicity, health insurance, smoking, hypertension, diabetes, hyperlipidemia, heart disease, and previous stroke history between those who could not identify any of stroke risk factor and those who could identify at least one.

Among all of the respondents, 17.7% perceived their risk for stroke, 49.8% believed that they had no risk for stroke, and 32.5% were unsure whether they had any risk for stroke. In 797 respondents aged 45 years or older, 19.8% believed they were at risk for stroke, and 46% did not perceive the risk for stroke. The results of the respondents’ perceived risk for stroke are shown in Table 1. Univariate analysis showed that gender, ethnicity, educational level, and household income had no effect on the respondents’ perceived risk for stroke (*P*>0.05). However, there was a difference in the perceived risk for stroke among different subject age groups (*P*<0.001). Respondents aged 45–64 years old were more likely to perceive their risk for stroke than other age groups. The perceived risk for stroke in the respondents with health insurance was slightly higher than those without health insurance (*P = *0.048). The proportion of respondents who perceived risk for stroke was significant higher in those with history of hypertension, hyperlipidemia, heart disease, and previous stroke than those without these stroke risk factors (*P*<0.001). Smoking and diabetes had no impact on the respondents’ level of perceived risk for stroke.

Multiple logistic regression analysis showed that 45–64 years age group, hypertension, hyperlipidemia, heart disease, and previous stroke history were independently associated with the perceived risk for stroke among all of the respondents (Table 1). For those with 2 or more stroke risk factors, only heart disease (adjusted odds ratio [AOR], 2.69; 95% Confidence Interval [CI], 1.48–4.90) and previous stroke history (AOR, 1.95; 95% CI, 1.03–3.70) were independently associated with the perceived risk for stroke.

**Figure 2:**
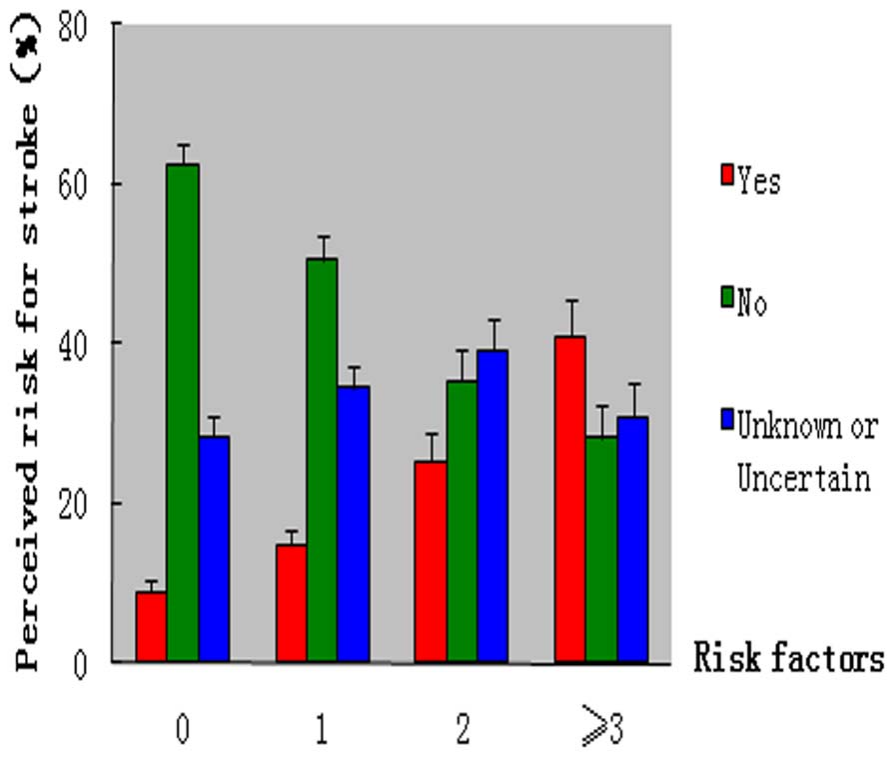
Stroke risk factors and perceived risk for stroke.

Overall, the perceived risk for stroke among the respondents with 0, 1, 2, and 3 or more stroke risk factors was 9.0% (95% CI, 6.0–12.0), 14.8% (95% CI, 10.9–18.8), 25.3% (95% CI,18.5–32.1), and 41% (95% CI, 32.1–49.9), respectively. The difference was statistically significant (*P*<0.001). (Figure 2).

In respondents aged 45 years or older, the perceived risk for stroke among those respondents with only 1 stroke risk factor was 16.5%. The perceived risk for stroke was higher in those aged 45–64 years than in those aged 65 years or older (20.9% vs. 10.1%, *P* = 0.019). Meanwhile, among respondents with 1 risk factor, the educational level of 45–64 years age group was significantly higher than ≥65 years age group (*P*<0.001). The perceived risk for stroke among the respondents with 2 or more stroke risk factors was 32%, but there was no significant difference in the perceived risk for stroke between those aged 45–64 years and those who were 65 years or older (34.4% vs. 29.8%, *P = *0.420).

In addition, among the respondents with only 1 stroke risk factor, the perceivable risk for stroke was 17.8% in those who could identify that this risk factor was a stroke risk factor, which was 10.7% (odds ratio [OR], 2.85; 95% CI, 1.16–6.99) higher than those who could not identify this risk factor. Among the respondents with 2 stroke risk factors, the perceivable risk for stroke was 37.7% in those who could identify that both risk factors were stroke risks, which was 26.6% (OR, 4.85; 95% CI, 1.06–22.11) higher than those who could not identify any of the two risks.

## Discussion

This community-based study found that 17.7% of the respondents perceived their risk for stroke. The proportion of respondents who perceived risk for stroke increased with the number of stroke risk factors increased, however, even among respondents with 3 or more risk factors, only 41% perceived risk for stroke. In addition, 45–64 years age group, hypertension, hyperlipidemia, heart disease, and previous stroke history were independently associated with the perceived risk for stroke.

We found most respondents with multiple risk factors did not perceive their risk for stroke. The results suggested that there was a serious lack of awareness regarding one’s own risk for stroke among the high-risk populations. Our results were similar to other results reported in the literature. Some studies have shown that the level of perceived risk for stroke was low in the elderly with stroke risk factors [10]. Samsa et al [11] surveyed high stroke risk populations in multiple medical centers in the United States and found that only 41% of the respondents perceived risk for stroke. The survey of Harwell et al [10] found that only 39% of the respondents in counties perceived risk for stroke. Gupta et al [12] conducted a survey in clinic patients with stroke risk factors who were over the age of 65 years and found that only 15% perceived their own risk for stroke. Our study found that among the participants who were over the age of 45 years, the perceived risk for stroke was 16.5% in those with 1 stroke risk factor, and 32% in those with 2 or more risk factors. Interestingly, some studies found that persons aged 45–64 years old were more likely to perceive themselves to be at risk for stroke compared to respondents aged 65 years or older [10]–[11]. We also found similar result in this study, and the reason may be related with higher educational level in 45–64 years age group. However, this difference only existed in the respondents with 1 risk factor; in respondents with 2 or more risk factors, there was no significant difference of perceived risk for stroke between the two age groups.

Study has found that being young, smoking, diabetes, hypertension, heart disease, hypercholesterolemia, and previous stroke/TIA were independently correlated with the perceived risk for stroke [10]. Similarly in our study, we also found that the perceived risk for stroke was independently associated with hypertension, hyperlipidemia, heart disease, and previous stroke history. Respondents with these risk factors may have more opportunity to receive health counseling from physician and therefore they were more likely to perceive risk for stroke. However, the perceived risk for stroke was not associated with smoking and history of diabetes. The reason may be related to the lack of awareness of these two stroke risk factors among respondents with diabetes and smoking. Our results indicated that the knowledge of the relationship between stroke risk factors and awareness of risk for stroke should be strengthen among those with stroke risk factors, especially among those with diabetes and smoking.

Our study found that compared with the respondents who had not awareness of stroke risk factors, those who could identify their stroke risk factors were significantly more likely to perceive themselves to be at risk for stroke. We noted that among the respondents with 1 and 2 stroke risk factors, the perceivable risk for stroke was only 17.8% and 37.7% respectively in those who could identify that their risk factors were stroke risk factors, which suggested that the lack of knowledge about stroke risk factors could not fully explain the low level of perceived risk for stroke. The reasons why most respondents with stroke risk factors denied the risk for stroke even though they could identify their risk factors were stroke risk factors remain to be elucidated. We speculate that one reason may be that some respondents think that only those with multiple stroke risk factors have risk for stroke. Actually even if there is only one stroke risk factor also means that there is presence of risk for stroke. The second reason may be that although having risk factors, some respondents think their diseases are not serious or in good control, and they are in good status of health, at least for now, so they feel stroke is far away from them or the occurrence of stroke is less likely. In addition, some respondents denied their risk for stroke due to social and psychological factors, for example, admitting there is a risk for stroke, a terrible disease, is likely to be stigma or loss of face.

Our study has some important implications about public health. At present, China’s stroke incidence and mortality of stroke continue to rise [3]. One of the most important reasons is that the control rate of stroke risk factors is very low. The persons with one or more stroke risk factors are targeted populations for stroke prevention. Since the perceived risk for stroke can promote the intervention of risk factors, the efforts of public health are much-needed to assure the populations with stroke risk factors, especially those with multiple risk factors clearly perceive their risk for stroke, which may prompt them to participate actively in the control of stroke risk factors. Some studies have suggested that health education regarding stroke that targets community residents could enhance the level of public awareness of stroke risk factors and warning symptoms [13], [20]–[24]. Therefore, similar health education which focuses on stroke prevention and risk awareness may help to improve the perceived risk for stroke among high-risk populations within communities. In addition, one study has identified that compared with high-risk patients who did not receive physician counseling, those who received counseling from a physician about their risk were more likely to perceive their risk for stroke [11], which suggested that it is useful to increase awareness of the risk for stroke in persons with stroke risk factors by the healthcare providers. Therefore, according to the patient’s individual information, healthcare providers should communicate with every patient with stroke risk factors to assure the patient’s definitely perceived risk for stroke, and make every patient understand why and how to control the stroke risk factors.

There are some limitations in this study. First, the survey was conducted in only three communities in Yuzhong district, so it might not reflect the situation of communities not been selected. Nevertheless, we could generally assess the level of perceived risk for stroke among Chongqing community residents through random sample. Secondly, the response rate was low despite prior notification and publicity in the communities, thus, there was a non-response bias in this cross-sectional survey. The characteristics of the persons who did not participate in the survey were not analyzed in this survey, so we didn’t know if the perceived risk for stroke among responding residents differ from those who refused to participate. We speculated several reasons may be related to the low response rate. Some community residents lack awareness of disease prevention and did not believe they would get any benefit from survey, so they might have no enthusiasm of participating in such survey. Some residents might worry about their privacy leakage if participating in such survey. In addition, stigma might have a role in the low response rate. We should further strengthen publicity work before survey, including explaining the purpose and significance of the survey especially the benefit from participating in the survey, and ensuring that all answers of questionnaire were anonymous and participating in this survey would not cause any bad influence. Moreover adding some incentive measures might help to reduce the refusal. Third, self-reported information regarding risk factors are subject to recall bias. Although we emphasized all of the diseases should be previously diagnosed through physical examination or/and assistant examination and the diseases information had ever been informed by a physician or nurse, nonetheless, it cannot be avoided because there were some differences of memory, concern for health and serious attitude to answer the questionnaire among respondents. Fourth, perception of risk for stroke is categoric (yes/no) in our study, but in fact respondents might perceive themselves at risk for stroke at any level. Thus, the survey results might have bias. It might be better that asking participants to rate their degree of risk for stroke according to their presence of risk factors. Last, the use of close-ended questionnaire might result in higher knowledge level of stroke risk factors than open-ended questions. However, the general residents’ knowledge of stroke risk factors can be reflected by using close-ended questionnaire.

In summary, this survey showed that awareness of stroke risk factors among the community residents in Chongqing is low and that there is a lack of perceived risk for stroke. Persons with 45–64 years old, a history of hypertension, hyperlipidemia, heart disease, or stroke were more likely to perceive risk for stroke. Therefore, medical personnel, including those in the communities, must take measures to improve the awareness of risk for stroke among the populations with stroke risk factors, thus contributing to the intervention of stroke risk factors and the reduction in the incidence of stroke.
